# CyberPsychological Computation on Social Community of Ubiquitous Learning

**DOI:** 10.1155/2015/812650

**Published:** 2015-10-19

**Authors:** Xuan Zhou, Genghui Dai, Shuang Huang, Xuemin Sun, Feng Hu, Hongzhi Hu, Mirjana Ivanović

**Affiliations:** ^1^School of Humanities and Social Science, Sichuan Conservatory of Music, Chengdu 610021, China; ^2^School of Marine Sciences, Sun Yat-Sen University, Guangzhou 510275, China; ^3^Overseas Training Center, Shanghai International Studies University, Shanghai 200083, China; ^4^Department of General Surgery, Tongji Hospital, Tongji University, Shanghai 200065, China; ^5^Department of Respiratory Medicine, Shanghai Tongren Hospital, Shanghai Jiao Tong University, Shanghai 200336, China; ^6^School of Management, Fudan University, Shanghai 200433, China; ^7^Department of Mathematics and Informatics, Faculty of Sciences, University of Novi Sad, 21000 Novi Sad, Serbia

## Abstract

Under the modern network environment, ubiquitous learning has been a popular way for people to study knowledge, exchange ideas, and share skills in the cyberspace. Existing research findings indicate that the learners' initiative and community cohesion play vital roles in the social communities of ubiquitous learning, and therefore how to stimulate the learners' interest and participation willingness so as to improve their enjoyable experiences in the learning process should be the primary consideration on this issue. This paper aims to explore an effective method to monitor the learners' psychological reactions based on their behavioral features in cyberspace and therefore provide useful references for adjusting the strategies in the learning process. In doing so, this paper firstly analyzes the psychological assessment of the learners' situations as well as their typical behavioral patterns and then discusses the relationship between the learners' psychological reactions and their observable features in cyberspace. Finally, this paper puts forward a CyberPsychological computation method to estimate the learners' psychological states online. Considering the diversity of learners' habitual behaviors in the reactions to their psychological changes, a BP-GA neural network is proposed for the computation based on their personalized behavioral patterns.

## 1. Introduction

In today's society, ubiquitous learning (U-learning) has been bringing about magical changes to traditional education [1–3]. Under the new learning patterns such as Webquest, ThinkQuest, Microlearning, and MOOCs (Massive Open Online Courses) [4–7], the learners can access online resources at any time, in any place, and through any multimedia terminal, and they can join an open community to study knowledge, exchange ideas, and share skills in cyberspace [4, 5].

In that circumstance, the participants of ubiquitous learning have formed an ecological social community which displays many new features and is sustained by the common motivation and goal, favorable atmosphere, and emotional communication, as well as enjoyable experiences [8, 9]. Many researchers have found that the effectiveness and sustainability of a social community of ubiquitous learning depend largely on the learners' initiative and the community cohesion, and therefore the most important question to be considered is how to stimulate the learners' interest and participation willingness, as well as improving their enjoyable experiences [8–10].

In the past century, learning theory has made great progress. Constructivism, situated cognition, and informal learning theories offer the guidance for ubiquitous learning by focusing on the individual's self-construction of knowledge and aim to provide new online intelligent, virtual interactive, and seamless learning patterns for learners on the basis of such situational characteristics as location, time, and environment [3, 8]. Research findings in neuroscience lay the basis of neuroeducation, which provides the neural theory and the brain mechanism for the study of ubiquitous learning [11]. In particular, recent work in this field has been well supported by the advanced technologies such as fMRI (functional magnetic resonance imaging), ERPs (event-related potentials), and DTI (diffusion tensor imaging). In particular, the blood oxygenation level dependent functional magnetic resonance imaging (Bold-fMRI) has been successfully applied to the neural activity studies of cognition and emotions in education [10, 12–16].

However, the environment and patterns of ubiquitous learning in a social community differ from those in classroom completely. Different social media and interactive activities between the participants have all major impacts on the cognition, emotion, and attitude in the learning process [8, 16–18]. In recent years, vocal social media such as Wechat, QQ (China), ICQ, WhatsApp (USA), and Line (Japan) and various tools of instant voice messaging have been the popular means in the communication of ubiquitous learning. While facilitating conveying semantic information, vocal social media can also transmit abundant emotional information [19]. This variation has resulted in significant influence on not only improving the participants' experiences and senses of belonging to particular social groups and therefore enhancing their continuance intentions to these groups [20], but also strengthening the interpersonal relationships between the members within these groups as well as the community's cohesion and cognitive consistence in this community [19, 21]. A new study shows that the interactive activities in a social community can cause the propagation effects on the five interactional layers, information, emotion, attitude, behavior, and culture, and easily lead to some groups of “the small world” with close relationships in the community [16, 19].

Therefore, considering the new features and special environment in cyberspace, a further study of ubiquitous learning theory is required to provide comprehensive and systemic guidance for improving the organizational mode, learning behaviors, and teaching skills, which involves the interdisciplinary areas of pedagogy, psychology, sociology, organizational behavior, neuroscience, and information technology. Scholars have paid attention to the above issue in the 1990s. CyberPsychology, coined by Dr. John Suler in his hypertext book “The Psychology of Cyberspace” with the first version appearing in January of 1996, launched the original conceptual framework for understanding how people react to and behave within cyberspace [22]. The progress in CyberPsychology and social neuroscience [23] has provided the fresh theory and new method for the development of computational intelligence in cyberspace.

By affective computing [24] on the learners' reactions and their behavioral data, the ubiquitous learning system may have emotional intelligence [25] and make the learning more “smart” which was defined by Professor Dai in 2012 as that the machine can perceive and respond to human emotional needs and provide the full humanized services combining both rational and emotional intelligence [26]. This concept has been applied in wide areas such as smart education, smart city, smart healthcare, and smart service [27, 28].

This paper aims to explore an effective method to monitor and analyze the learners' psychological reactions based on their observable behaviors in cyberspace and therefore help to conduct the smart education in ubiquitous learning. This paper is organized as follows.  Section 1 is an introduction to the research background and motivation.  Section 2 discusses the research model. The CyberPsychological computation method is put forward in Section 3 with its experiment and result in Section 4.  Section 5 is the conclusion and discussion.

## 2. Psychological Assessment and Behavioral Patterns

### 2.1. Psychological Assessment of Learners' Situations

Social community of ubiquitous learning is affected by a lot of factors associated with social, psychological, organizational, managerial, and technological aspects. Since the 1990s, a lot of scholars such as Webster and Hackley [29], Hill and Hannafin [30], and Hannafin et al. [31] have studied the influence factors and cognitive characteristics related to ubiquitous learning environment. In the late research, Chinese scholar concluded the influence factors as a LICE (Learner, Instructor, Curriculum, and Environment) model [32].

Recent experimental observations showed that a desirable learning atmosphere, good visual effects, pleasant voices, suitable topics and materials, and positive evaluation feedbacks were the most important factors that aroused the learners' interest in ubiquitous learning and contributed to the pleasant emotional experiences [10]. In order to adjust the strategies dynamically, the learners' psychological reactions such as attention, interest, emotion, and satisfaction are usually applied as the monitoring variables in the learning process [17]. Although the above four variables are actually not independent, the learners can get clear understanding of them and make accurate subjective assessment on each variable. Therefore, the dynamically psychological assessment of learners' situations can be expressed as formula

(1) PAti=Ati,Iti,Eti,Sti,i=1,2,3,…,N,

where PA(*t*_*i*_) represents the vector of psychological assessment at different times *t*_*i*_  (*i* = 1,2, 3,…, *N*) given by the learners and *A*(*t*_*i*_), *I*(*t*_*i*_), *E*(*t*_*i*_), and *S*(*t*_*i*_) represent the scores of attention, interest, emotion, and satisfaction, respectively, at time *t*_*i*_.

The calibration for scoring records is shown as in Table 1. Here, attention and interest are scaled from 0 to 10 with the intensity varying from low to strong, but emotion and satisfaction are scaled from −5 to +5 with the polarity and strength varying from the most negative to the extremely positive.

### 2.2. Behavioral Patterns in Learning Process

In ubiquitous learning, the learner's behaviors can be divided into three categories from simple to complex: operational behaviors, information exchanging behaviors, and problem-solving behaviors [33]. However, psychological reactions of the learners are mostly reflected in their habitual behaviors which are composed of a series of basic actions in cyberspace [8].

The pattern of each learner's habitual behaviors can be expressed as formula

(2) $$\begin{array}{c} BP\left(\;p_{a}\;\right)=\left\{\;A\left(\;j\;\right),P\left(\;j\;\right)\;\right\},\;j=1,2,3,\ldots ,M, \end{array}$$

where BP(*p*_*a*_) represents the behavioral pattern related to psychological reaction *p*_*a*_, *A*(*j*)  (*j* = 1,2, 3,…, *M*) represents a series of basic actions in a certain period *T* under the psychological reaction *p*_*a*_, and *P*(*j*)  (*j* = 1,2, 3,…, *M*) is the parameters of *A*(*j*).

The action's data are mainly acquired from the server's log files or by some online tracking tools. The switching frequency and retention time of webpages, the locations and movements of the mouse, and the keyboard operations are all the important parameters as the features to reflect the learners' psychological reactions. For example, if the learner has strong interest in something and is in a fairly good mood, he/she tends to stay on the interesting webpage longer, use his/her mouse and keyboard with higher frequency, answer the asked questions more quickly, and be more willing to make positive comments. On the contrary, if he/she is anxious and fretful, he/she will switch from one webpage to another frequently, move the mouse in a wide range quickly, and be more likely to give negative feedback [10]. Professor Dai proposed a CPP (CyberPsychological and Physical) computation method based on social neuromechanism and concluded 15 commonly basic actions of the user's habitual behaviors in cyberspace as in Table 2 [34].

### 2.3. Behavioral Features in Cyberspace

The psychological reactions of the learners are the results of a series of neural activities dominated by the brain mechanism [8], which will not only generate activated responses in their brains, but also result in the corresponding variation of physiological signals (e.g., EEG, ECG, EDR, respiration, and skin temperature) as well as external performances (e.g., speeches, facial expressions, gestures, and movements) and the possible subsequent behaviors [16, 23, 35, 36]. Therefore, the psychological computation in a real environment can be conducted by analyzing the expression patterns on the reactions of physiological signals, external performances, and subsequent behaviors based on the fusion features corresponding to the certain reactions in brain areas [10, 13, 26].

However, in the ubiquitous learning environment, the possible information we can obtain from the learners is their observable actions as well as the voice and video signals produced by their online activities under some circumstances. Considering the protection of the learners' privacy, CyberPsychological computation cannot be based on the content analysis and semantic detection of the above information. Fortunately, technologies of psychological computation from voice and video signals have developed quickly in recent years. For example, progress has been made on the voice signal based on its acoustic features parameters such as speech speed, voice intensity, pitch frequency, LPCC (Linear Prediction Cepstrum Coefficient), and MFCC (Mel Frequency Cepstrum Coefficient) [19, 37, 38].

**Figure 1:**
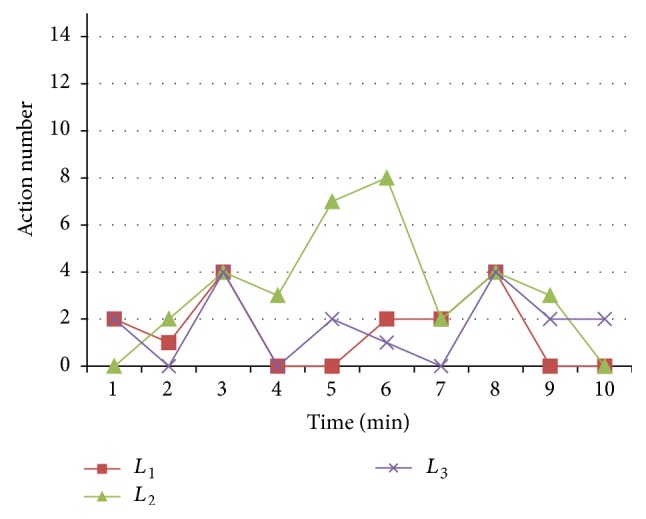
Actions of three learners to the same psychological reaction PA{9.3,9.2,2.5,9.1} in a period of 10 minutes.

In our research, we mainly consider the learners' behavioral features from their observable actions in cyberspace. The relationship between the learners' psychological reactions and their observable features is related to the habitual patterns of the learners' behaviors, which is dependent on a statistical study in the real cases.  Figure 1 shows the actions of three learners (*L*_1_, *L*_2_, and *L*_3_) to almost the same psychological reaction PA{9.3,9.2,2.5,9.1} (within the relative errors of 10%) in a period of 10 minutes.

From Figure 1, we can find most of the learners' actions are Action number 2 and Action number 4, but there is diversity in the action's orders and frequencies for different learners, which indicates that the CyberPsychological computation should be based on the learners' personalized behavioral patterns.

## 3. CyberPsychological Computation Method and Technology

### 3.1. Computation Method

**Figure 2:**
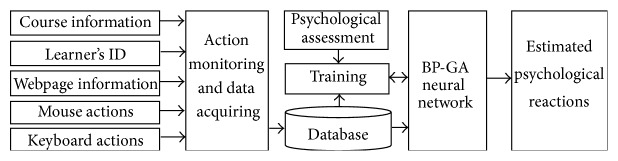
CyberPsychological computation method on social community of ubiquitous learning.

In order to monitor the learners' psychological reactions in the learning process, we put forward a CyberPsychological computation method as shown in Figure 2. The course information, learner's ID, and webpage information as well as the actions of mouse and keyboard are monitored and acquired by the computation system online. After preprocessing, those data are stored in a database for training or computation by a BP-GA neural network.

In the learning system, online activities such as answering questions, retrieving information, discussing problems, chatting with each other, and submitting assignments are all assigned in different functional areas on the webpage. So the layout structure and its related information of the webpage are extracted to assist in identifying the learners' action.

Every change of the elements on the webpage, such as moving, clicking, and scrolling of the mouse or the operation of the keyboard, will trigger the corresponding JavaScript function. In order to meet the requirements of real-time data collection, we adopted the PHP language to program the above JavaScript function and process the mouse and keyboard data acquisition, which can be realized by the intelligent multiagent technology [10].

As we discussed before in this paper, the learners' psychological reactions are exhibited in their diverse habitual behaviors and the CyberPsychological computation should be based on their personalized behavioral patterns. Therefore, we consider the learner's ID and his/her historical behavior patterns in our method. The computation on the learners' psychological reactions based on their behavioral features in the learning process can be regarded as a dynamic and nonlinear estimation problem by machine learning. So the subjective psychological assessment of learners' situations should be given by them in the sample training.

Nonlinear Regression, Kalman Filter, Artificial Neural Network (ANN), and SVR (Support Vector Regression) have been reported as successful technologies for solving the above estimating problem [10, 19]. However, the relationship between the learners' psychological reactions and their observable features in cyberspace is affected by many factors. BP neural network has the advantages of no specific requirements on the data distribution and no sensitivity to the influence of multicollinearity and outlier data and can be trained by increasing samples to reveal the implied relationship between input and output variables more comprehensively and obtain higher estimation precision, so it is appropriate to solve this problem. For the purpose of accelerating the convergence rate as well as improving the generalization ability of computation [39, 40], we adopt the technology of BP-GA neural network in our method.

### 3.2. BP-GA Neural Network

BP (back propagation) neural network is a nonlinear function to establish the uncertain and continuous relations between input and output variables based on trained samples by machine learning. It can continuously modify the network weights and thresholds through the error back propagation algorithm (BP algorithm) and reach the target of minimizing the mean square error. However, the traditional BP neural network easily causes the nonconvergence problem or falls into a local extremum. Those defects can be overcome by combining with a genetic algorithm (GA) [39, 40].

**Figure 3:**
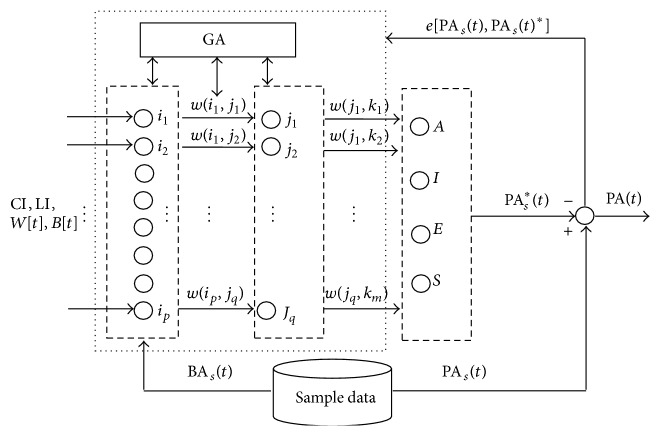
BP-GA neural network for CyberPsychological computation.

Figure 3 shows the proposed BP-GA neural network for CyberPsychological computation in our method. A3-layer BP neural network is mainly used to refine the generalized relationships between the input and output variables and produce the estimated psychological reactions of the learners based on their observable features in cyberspace. In this computation, GA contributes to the acceleration of convergence rate and prevents the network from going down to local minimum points by optimizing the weights and thresholds of the BP network.

The input layer has 12 nodes which are composed of the data of course information, learner's ID, mouse actions, and keyboard actions. The output layer has four nodes which correspond to the scores of the learner's psychological reactions in formula (1). The hidden layer is set with 22 nodes according to our best test result.

The BP-GA neural network operates firstly with the training by a group of sample data which are tested and recorded in the real cases. After it converges to a stable state, the BP-GA neural network can be applied to the computation in the learning process.

## 4. Experiment and Result

**Figure 4:**
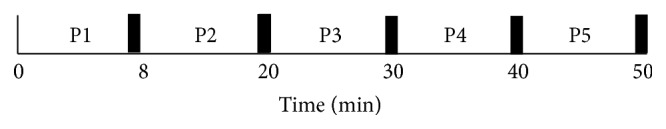
Learning process in each lecture of training course of life health and medical emergency rescue.

The experiment is based on a training course of life health and medical emergency rescue. In order to exclude the effects of the instructor, we edited this course into 20 lectures which are all taught by video tutorials and operated under a designed controlling program in the learning process. Every lecture runs for 50 minutes and is divided into 5 periods (from P1 to P5) with different activities arranged in each period.  Figure 4 shows the design of learning process in each lecture of this course.

The learners are 16 social participants with more than 60 learning hours in a normal and stable environment of ubiquitous learning community. They are required to complete this course in 40 days. However, they can arrange the learning time freely in a ubiquitous learning environment. In this experiment, we assigned different activities in each period of a lecture and asked the learners to make a psychological assessment of their general situations in the final 1 minute of each period.

Table 3 shows the duration and activities in each period of a lecture.

In the end of our experiment, 11 participants successfully finished this course and provided completed subjective assessments of their psychological reactions in the learning process. We extracted randomly the records of 16 lectures as the training samples for BP-GA neural network and took the rest of the 4 lectures for the test of computation results. The above process was carried out 3 times.  Table 4 is the test results of CyberPsychological computation.

Table 4 shows that estimated relative errors of the learners' attention, interest, emotion, and satisfaction are −15.4%, 6.0%, 22.3%, and 17.1%, respectively. It indicates that our method can achieve an accuracy near 78% which can provide effective monitoring of the learners' psychological reactions in a real ubiquitous learning environment. From Table 4, we can find that the errors of attention, emotion, and satisfaction are larger than that of the interest. This is because the learner may have more actions to look for the further details of his/her interesting contents in this state. Also, the standard deviations of attention, emotion, and satisfaction are larger than the interest due to possibly less actions in the above states, which should cause more deviation errors in the computation because of lesser observation data. However, the computational accuracy can be improved markedly if an interactive activity is introduced. For example, the system may suggest a response to be made by the learner who has lesser actions by asking him/her a question or letting him/her to discuss with the other learners.

By analyzing learners' psychological reactions and their varying characteristics in the learning process, we can explore the statistical distribution and the changing rhythms of the learning community in different periods and scenarios and find the learners' ROI (Region of Interest). This has significant implications on the design of learning strategies such as the education scheme and teaching plans which are on-demand and more appealing and enjoyable to learners in order to provide state-of-the-art teaching models, skills, and technologies for smart education in a ubiquitous learning environment [10].

## 5. Conclusion and Discussion

With the development of modern information network and mobile communication technology, personalized learning in a ubiquitous learning environment has been made possible in web space accessible by a variety of multimedia terminals anytime and anywhere. It has brought about new changes to the theories, means, and patterns of traditional education. In the U-learning environment, learners' psychological reactions and learning experiences have significant impacts on stimulating their learning interest and improving the teaching efficiency.

This paper aims to explore an effective method to monitor the learners' psychological reactions based on their behavioral features in cyberspace and therefore provide useful references for adjusting the strategies in the learning process. As the comprehensive result of our research, a CyberPsychological computation method based on BP-GA neural network was proposed for estimating the learners' psychological states online. The experimental result shows that it can achieve accuracy near 78%.

Future researches should consider the dynamic psychological reactions of the learners through the studies of their physiological signals such as EEG, ECG, EDR, respiration, and skin temperature by a wearable device system and assist in obtaining a more precise psychological assessment of the learners' situations. Besides, the influence factors of different lecture's contents and the statistical distribution of the learning community as well as its varying characteristics in the learning process are all worthy of further explorations based on more cases and samples.

## Figures and Tables

**Table 1:** Calibration for scoring records in psychological assessment.

Variables	Scores										
Attention	0	1	2	3	4	5	6	7	8	9	10
Interest	0	1	2	3	4	5	6	7	8	9	10
Emotion	−5	−4	−3	−2	−1	0	1	2	3	4	5
Satisfaction	−5	−4	−3	−2	−1	0	1	2	3	4	5

**Table 2:** Basic actions and parameters of the learner's habitual behaviors in cyberspace.

Number	Actions	Parameters
0	No action	The object in screen center
1	Mouse: click	On a button or link, on another place
2	Mouse: scroll	Speed, the object in screen center when stopping scrolling
3	Mouse: move	Speed, radius
4	Mouse: open a new page	Null
5	Mouse: change a page	Null
6	Mouse: close a page	Null
7	Mouse: store a page	Null
8	Keyboard: input	Number of characters
9	Keyboard: delete	Number of characters
10	Mouse and keyboard: retrieve information	Number of keywords
11	Mouse and keyboard: post information on BBS	Number of characters
12	Mouse and keyboard: send information to other people	Number of characters, number of receivers
13	Mouse and keyboard: chat with other people	Number of characters, number of people chatted with
14	Streaming media: voice communication	Acoustic feature parameters
15	Streaming media: video communication	Visual feature parameters

**Table 3:** Duration and activities in each period of a lecture.

Period	Duration	Activities
P1	8	Watching video tutorials
P2	12	Watching video tutorials
P3	10	Watching video tutorials
P4	10	Completing an online individual assignment
P5	10	Discussing with other learners

**Table 4:** Test results of CyberPsychological computation.

Variable	Average score by assessment	Estimated result by computation	Relative error	Standard deviation
*A*	7.213	6.102	−15.4%	1.237
*I*	7.862	8.333	6.0%	0.6752
*E*	4.220	3.281	22.3%	1.401
*S*	8.581	7.114	17.1%	1.898
